# From communities to protein complexes: A local community detection algorithm on PPI networks

**DOI:** 10.1371/journal.pone.0260484

**Published:** 2022-01-27

**Authors:** Saharnaz Dilmaghani, Matthias R. Brust, Carlos H. C. Ribeiro, Emmanuel Kieffer, Grégoire Danoy, Pascal Bouvry

**Affiliations:** 1 Interdisciplinary Centre for Security, Reliability and Trust (SnT), University of Luxembourg, Esch-sur-Alzette, Luxembourg; 2 Computer Science Division, Aeronautics Institute of Technology (ITA), São Josédos Campos, Brazil; 3 Faculty of Science, Technology and Medicine (FSTM), University of Luxembourg, Esch-sur-Alzette, Luxembourg; Unviersity of Burgundy, FRANCE

## Abstract

Identifying protein complexes in protein-protein interaction (ppi) networks is often handled as a community detection problem, with algorithms generally relying exclusively on the network topology for discovering a solution. The advancement of experimental techniques on ppi has motivated the generation of many Gene Ontology (go) databases. Incorporating the functionality extracted from go with the topological properties from the underlying ppi network yield a novel approach to identify protein complexes. Additionally, most of the existing algorithms use global measures that operate on the entire network to identify communities. The result of using global metrics are large communities that are often not correlated with the functionality of the proteins. Moreover, ppi network analysis shows that most of the biological functions possibly lie between local neighbours in ppi networks, which are not identifiable with global metrics. In this paper, we propose a local community detection algorithm, (lcda-go), that uniquely exploits information of functionality from go combined with the network topology. lcda-go identifies the community of each protein based on the topological and functional knowledge acquired solely from the local neighbour proteins within the ppi network. Experimental results using the Krogan dataset demonstrate that our algorithm outperforms in most cases state-of-the-art approaches in assessment based on *Precision*, *Sensitivity*, and particularly *Composite Score*. We also deployed lcda, the local-topology based precursor of lcda-go, to compare with a similar state-of-the-art approach that exclusively incorporates topological information of ppi networks for community detection. In addition to the high quality of the results, one main advantage of lcda-go is its low computational time complexity.

## Introduction

Proteins work cooperatively to accomplish biological functions. The physical interaction between proteins, known as *protein-protein interaction* (ppi), is the key for many biological functions [1], for example, the transcription of DNA, the translation of mRNA, and cell cycle [2]. Scientific progress on ppi is highly critical for applications such as protein function discovery [3], disease comprehension [4], and drug discovery [5].

To infer the physical interactions of proteins, a number of experimental techniques have been developed, such as *yeast-two-hybrid* (y2h) [6] and *tandem affinity purification* (tap) [7]. This resulted in the generation of several depositories and databases of experimental data on ppi (e.g., biogrid). While these screening methods facilitate the comprehension of ppi, they have been widely criticized due to the false negative (i.e., not being able to detect interacting proteins) and false positive (i.e., identifying non-interacting proteins as interacting proteins) interaction detection. Therefore, high-throughput screening methods suffer from a considerable lack of accuracy and thus, produce an incomplete map of the interactions among the proteins [8–10].

The pairwise physical interaction of proteins within the ppi data suggests a network representation where nodes are the proteins and links are the interactions among the proteins. Exploiting network structure with network analysis tools on such data has shifted the ppi analysis to the *network* level. Besides, the existence of protein complexes justifies the high-degree clusters within the ppi network [9]. ppi networks inherit both *topological* and *functional* information [1]. The first term refers to the physical interaction describing the arrangements of the nodes in the network, and is associated with the densely connected proteins namely *communities*. The latter explains the biological function of proteins that are achieved by groups of proteins that bind each other and shape *protein complexes*. The complexes are explained by the annotations available in Gene Ontology (go) [11, 12] databases. go provides a specific definition of protein functions and it is one of the main resources of biological information. go provides a structured and controlled vocabulary of terms, which are subdivided into three non-overlapping ontologies: Molecular Function (mf), Biological Process (bp) and Cellular Component (cc) [13]. We utilize go terms to enrich ppi networks with functional properties of proteins.

It is acknowledged that in several cases, those proteins that are topologically interconnected represent similar biological processes (i.e., go terms) [14], thereby there is an overlap between the communities of proteins and complexes. Nevertheless, the two terms are distinguished entities in ppi networks. Moreover, biological networks such as ppi networks share a common feature refereed as *local cluster connectivity* [15] that highlights the locality of the biological functions in ppi networks that are possible only between local neighbours.

Because of the correlation that exists between protein communities and complexes, detecting protein complexes from ppi networks can be translated into a community detection problem [2, 16, 17]. The purpose of a community detection algorithm for ppi networks is to divide proteins into groups such that the proteins of the same group are more similar to each other rather than those in the other groups. The state-of-the-art solutions consider different objectives to divide the nodes of a given network into highly interconnected communities [18–20]. Some of these algorithms are adjusted to biological networks to tackle the protein complex detection in ppi networks [21], including c-finder, coach, ClusterOne, mcl, cmc, mcode, and core&peel. Even though the community detection algorithms drive optimal topological communities in ppi networks, they suffer from the particular biological nature of the network due to the disengagement of functional properties. [2, 10, 22, 23].

The extracted interactions from experimental techniques (e.g., y2h, tap) are sometimes biased with incorrect inferring of existing and non-existing relationships. In other words, the available ppi networks could be incomplete and unreliable with respect to the detected nodes and links [9]. That in return will impact the results of the communities if the method depends solely on the existing topology of the network [24]. Moreover, some of the existing community detection algorithms acquire the whole network, that could be inherently incomplete, and hence results in large tangled communities of mixed or broad functionality [25] that do not explain adequately the underlying ppi network [23, 26]. In addition, such algorithms perform based on the global measures that are expensive in time complexity.

Encoding biological information in ppi networks could address the challenge of detecting higher quality communities of proteins with respect to their biological nature. The functionality hence could be achieved by incorporating biological information from the annotated databases (e.g., go, david). dcafp [27], gmftp [28], and mtgo [23] are some of the algorithms that are designed in a similar way. To tackle the next challenge regarding the reliability of the data and missing information, one possible solution could be to diminish the impact of network structure by focusing only on the local neighbours [29].

In this paper, we propose lcda-go, a local community detection algorithm that combines topological and functional properties (i.e., go terms) of ppi networks to detect associated communities that are representing protein complexes. One of the main advantages of lcda-go is the strong degree of locality [30] devised in the algorithm which not only reduces the dependency to the network structure but also equips the algorithm with a considerably low time complexity when compared to other state-of-the-art approaches. We compare lcda-go with the state-of-the-art algorithm that incorporates the topology and functionalities by exploiting go to detect protein complexes. We also expand our experiments by providing a comparative evaluation with state-of-the-art protein complex detection approaches relying only on the topology of the network. For this experiment, we have used the lcda algorithm [29], the local-topology based precursor of lcda-go.

## Related work

Many algorithms have been proposed to detect communities in ppi networks [2, 21, 31, 32]. Some of these approaches just rely on the topology of the ppi networks to detect communities, while others combine the biological functionality of the nodes to enrich the network and hence complex detection. We classify the existing community detection algorithms used for protein complexes in two categories based on the properties that an algorithm incorporates to detect the communities. We first explain community detection algorithms that perform solely on the *topology* of a network, and then, we discuss algorithms that rely on both *topology* and *functionality*.

### Topological approaches

One of the earliest algorithms that has been developed for ppi networks community detection is mcode [33]. It enjoys a level of locality, by expanding a set of high-ranked nodes (i.e., source nodes) into communities. mcode often represents very large communities and hence the number of predicted real complexes is small. The Markov Cluster algorithm (mcl) [34] is also utilized on ppi networks. The algorithm is a robust method based on a random walk to partition the network into communities. ClusterOne is a greedy approach starting from a seed node. The nodes with high cohesiveness are added or removed from the communities in an iterative process. ClusterOne is an overlapping community detection approach and it merges those groups of proteins that satisfy an overlap score.

For the comparative evaluation we used mcode, mcl, and ClusterOne [35] to measure the performance differences of our lcda algorithm, a version of lcda-go performing based on just local topological properties. Other algorithms such as coach [36] and lcma [37], and CFinder [38] also benefit from topology of the network to find the communities. These algorithms are discussed in [2, 21, 31].

### Topological and functional approaches

Recent approaches benefit from functional enrichment of the network to accurately detect the communities of proteins in ppi networks. The main motivation of such algorithms lies in the fact that protein complexes are mostly aggregated in performing common functions. One of the earliest approaches in this category is rnsc [39]. This algorithm is initialized with a random partitioning that is optimized based on the minimum cost for node exchanging. It considers density and functional homogeneity to search for better communities. Its performance, however, depends on the initial community assignment. mtgo [23] is a recent approach that combines both topological and functionality of the ppi networks to detect the communities. Similarly to rnsc, mtgo initializes the process by a random partitioning, and decides on rejoining the nodes into the communities if they share a common functionality and also if the new node increases the modularity of the community. The algorithm relies on two parameters *min* and *max* that control the size of the communities and impact the outcome. gmftp [28] and dcafp [27] are two other algorithms that are designed similarly by exploiting functionality, however, the biological nature of the networks are not directly involved in the main process and it is rather processed in advance by the network topology.

Our proposed lcda-go approach is similar to mentioned algorithms such that it combines both topological and functional information. However, unlike rnsc, mtgo, our proposed model does not rely on any random partitioning nor is restricted to initial input parameters. The results of lcda-go is compared to mtgo in Experiments and Results Section.

## Local Community Detection Algorithm for protein complexes with Gene Ontology (LCDA-GO)

In this section, we introduce the basic notation and terminologies that will be used through the paper. We also describe how lcda-go is implemented to detect communities of proteins exploiting topological and functional properties based on local conditional rules.

### Notation and Preliminaries

We assume an undirected and unweighted network *G* = (*V*, *E*), where *V* and *E* represent the set of nodes and the set of links, respectively. Our purpose is to divide *G* into set of communities, *C*, such that each node *v* ∈ *V* belongs exclusively to one community *c*_*i*_, and *C* = ⋃*c*_*i*_. A high quality community is a densely intra-connected (topology property) group of proteins representing lowest variation of go terms (functional property). lcda-go finds communities based on both topological and functional properties in a local manner. The algorithm allows each node to adjust its community label, *cl*, given the local neighbourhoods.

**Fig 1:**
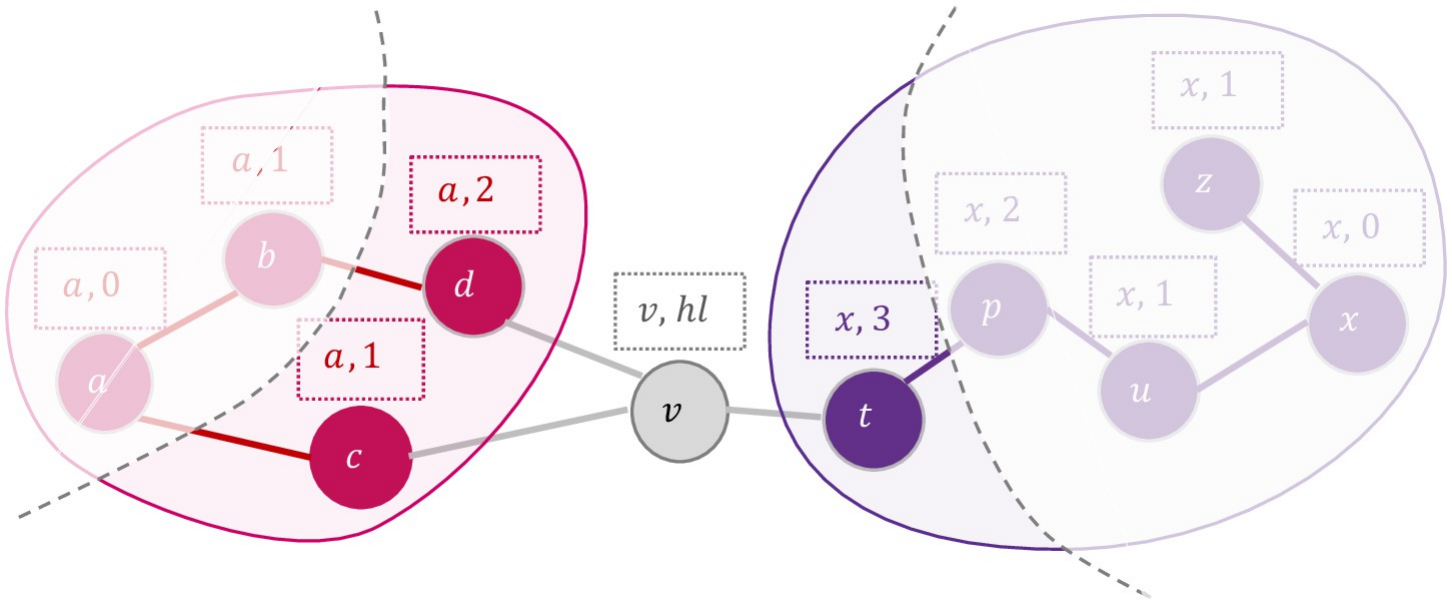
A snapshot of the community structures and local information that lcda-go is implemented on for node *v*. The transparent area is unknown zone that is not available during the operations. Thus, each node performs relying on the knowledge of its first neighbours. In this example, *c* and *d* are from community *a* and *t* is in community *x*. The community label describes the source node of the community, hence, *a* and *x* are two surrounded communities of *v*. The numbers attached to each node describes the hop-distance of the node from its community presenter. During the implementation, we have considered *hl* of a source node equal to 1 instead of 0.

On a given ppi network, lcda-go represents communities by a source node that is discovered during the algorithm. A source node is one of the high-degree nodes of the community and is connected to the nodes that have similar functional properties. The distance from the source node of a community to node *v* is stored in *hl*_*v*_. A snapshot of lcda-go performance is illustrated in Fig 1 showing the process for node *v*. In this scenario, *v* has three neighbours [*c*, *d*, *t*], such that node *c* and *d* belong to ‘*a*’ and *t* is from *x* (i.e., *cl*_*c*_ = ‘*a*’, *cl*_*d*_ = ‘*a*’, *cl*_*t*_ = ‘*x*’). Besides, the numbers show *hl* of each node, that is the hop-distance from the source node of the community. According to this example node *c* and node *d* are 1 and 2 hops away from the source node of their community (i.e., *a*), respectively, and *t* is 3 hops away from its source node, *x*. It is worth mentioning that *v* does not have any other knowledge about the rest of the network as shown in the transparent zone in Fig 1.

Besides the above-mentioned topological variables, *cl* and *hl*, that are consider in lcda-go, *g* is also determined to store go terms that a protein is contributed. To access a decision on the community of node *v*, lcda-go calculates two parameters as defined in the following:

**Definition 1**. *(Community influence degree.)* The community influence degree of node *v* is calculated between *v* and its neighbours from community *c*_*i*_ as follows:

$$\begin{array}{c} \lambda {\left(v\right)}^{u\in [\Gamma \left(v\right)\cap c_{i}]}=\text{ln}\left(\frac{k_{v}}{hl_{v}}\right).\left|g_{v}\cap g_{u\in [\Gamma \left(v\right)\cap c_{i}]}\right|, \end{array}$$ (1)

where $|g_{v}\cap g_{u\in c_{i}}|$ is the number of common go functions between *v* and its neighbours from community *c*_*i*_. The intuition behind the community influence degree is that a node is more likely to be in the same community as a neighbour node if the following node is closer to the source node of the community, has a higher degree, and shares similar functions with the neighbour node. If in a community one node has a higher community influence degree, the node could be a potential source node.

**Definition 2**. *(Local community modularity.)* The local community modularity for a node *v* is calculate for a surrounded community *c*_*i*_ as:

$$\begin{array}{cc} \mu {\left(v\right)}^{c_{i}}= & \frac{E_{in}-E_{out}}{E_{in}+E_{out}}\\ = & 2\frac{E_{in}}{E_{in}+E_{out}}-1, \end{array}$$ (2)

where *E*_*in*_ is the number of links connecting node *v* to nodes from community *c*_*i*_, and *E*_*out*_ represents the links to the other nodes. The value of local community modularity can vary in the range of (−1, 1]. It takes a negative value if there is no link to community *c*_*i*_. The value is positive if the number of links connected to *c*_*i*_ surpasses the number of links to other communities. Local community modularity performs as a measure of community extension by adding *v* to *c*_*i*_, if $\mu_{v}^{c_{i}}$ is positive.

A list of the notations used in the paper is summarized in Table 1.

**Table 1:** Notation exploited in lcda-go.

*G*	A ppi network
*C*	Set of solution that consists of communities of *c*_*i*_ such that *C* = ⋃*c*_*i*_
*v*	The current node
Γ(*v*)	Neighbours of node *v*
*k*(*v*)	Degree of node *v*
*cl*(*v*)	Community label of *v*
*hl*(*v*)	Hop-distance from the community source node
*g*(*v*)	go terms of node *v* (i.e., functional properties)
λ(*v*)	Community influence degree on node *v*
*μ*(*v*)	Local community modularity

### Algorithm description

We propose an iterative bottom-up approach, lcda-go, allowing each node to take a decision of joining a community independently. Our algorithm starts from a node and discovers the network through each node’s direct neighbours. lcda-go relies on a set of conditional rules to expand or generate new communities. The Local Community Expansion Rules (lcer) operate on each node based on the acquired local neighbourhood information as explained in Notation and Preliminaries Subsection. At each step of lcda-go nodes adjust their hop-distance (*hl*) value according to their distance from source nodes. If a node has a higher community influence degree and meets the conditions, it will become a source node. Thus, its *hl* is updated to 1. In this case, all neighbour nodes adjust their *hl* according to their hop-distance from the source node. lcda-go converges when all nodes agree with their community labels. A pseudo code of the proposed lcda-go is described in Alg. 1 lcda-go. The algorithm starts by initializing the node list *R* (line 1), that records the visited nodes and their neighbours. The initial node is either a given node or randomly selected from the network. As a first-time-visited node in the list, the community label *cl* of the node is assumed as it ID, in this case, v, and its hop-distance *hl* is set to the constant value of *HL* (line 2-3). We chose *HL* = 4 initially, however, it can be any value larger than 1. The next step is to adjust *v*.*hl*: If *v*.*hl* is the highest compared to *v*’s neighbours, then it will be reduced by 1 (lines 7-8). Afterwards, λ(*v*) and *μ*(*v*) is calculated (lines 10-11) and *v* is transmitted to Alg. 2 lcer (line 12) to make a decision regarding its *cl*. employing lcer on *v*, its attributes such as *cl* and *hl* will be updated consequently. Next, *R* expands by including neighbours of *v*. The processes continue such that all nodes of *V* is included in *R* and updated by lcer. Finally, if all nodes come to an agreement such that no further changes occur in community structure and each node of the network is declared in one community, the algorithm will converge. The stopCondition is defined as follows:

$$\begin{array}{c} \text{stopCondition}=\{\begin{array}{cc} 1, & \text{if}\;(R==V)\;\&\;\text{(for}\;v\;\text{in}\;R\text{,}\;v.cl\;\text{doesn}’\text{t}\;\text{change)}\\ 0, & \text{otherwise.} \end{array} \end{array}$$ (3)

After the convergence of lcda-go, the set of communities is obtained by retrieving each node’s *cl* from *R*.

**Algorithm 1** lcda-go

**Input**: Network *G*

**Output**: *C* set of communities

 *Initialisation*:

1: *R* ← *v* from *V*

2: *v*.*hl* = *HL*

3: *v*.*cl* = v

4: *v*.*g* = GO[*v*]

 *Procedure*:

5: **while** stopCondition **do**

6:  **for** *v* in *R* **do**

7:   **if** *k*_*v*_ > max(*k*_Γ(*v*)_) **then**

8:    *v*.*hl* ← *v*.*hl* − 1

9:   **end if**

10:   *v*.λ = λ(*v*)

11:   *v*.*μ* = *μ*(*v*)

12:   lcer(*v*)

13:   *R*.update ← Γ(*v*)

14:  **end for**

15: **end while**

16: **return** *C*.update ← *cl* from nodes of *R*

We defined Alg. 2 lcer to decide the corresponding community of *v*. For an input node *v*, it first calculates the local community modularity. Instead of computing the function for each *c*_*i*_, we only consider the larger community(ies) which has the larger number of links to *v*. We assume that u is the larger community. If *μ*(*v*) is positive, *v* joins community u. Thus, the community label of *v* changes to u (line 3), and the hop-distance shift to the shortest path from *v* to the source node *u* (line 4). To measure the shortest path, we simply consider the minimum *hl* of the neighbours plus 1. In case *μ*(*v*) is negative or zero, one of these two scenario may occur: First, the algorithm checks for the possibility of *v* itself being a source node. It means that node *v* is selected by the neighbours as the source node, while its attributes are not updated. Hence, the attributes of *v* are changed to fit the condition (line 7-8). Otherwise, *v* changes its attributes to follow the most similar node in its neighbourhood, which is node *p* with highest community influence degree (line 9-10). then, either *v* itself is selected by the neighbours to be a new community, or it will temporarily follow the best candidate among its neighbourhoods.

**Algorithm 2** lcer

1: **if** (*μ*(*v*) > 0) **then**

2:  *v*.*hl* = min(Γ(*v*).*hl*) + 1

3:  *v*.*cl* = u

4: **else if** (*μ*(*v*) <= 0) **then**

5:  **if** *v*.*cl is* u **then**

6:   *v*.*hl* = 1

7:   *v*.*cl* = u

8:  **else**

9:   *v*.*hl* = *p*.*hl*

10:   *v*.*cl* = *p*.*cl*

11:  **end if**

12: **end if**

### Computational complexity

The complexity of the proposed algorithm is determined by two loops in the algorithms. The outer *while*-loop in Alg. 1 lcda-go—line 5 coordinates the convergence of lcda-go to ensure that all nodes have come to an agreement about their community assignments. The recurrence (*t*) of the outer loop is independent from the size of the network. Our experiments with various networks’ sizes [29] shows that 8 ≤ *t* ≤ 15. The inner *for*-loop of lcda-go described in 1 line 6, operates a set of conditional rules over each node from list *R*. The performance of the inner loop has the highest impact on the overall complexity of lcda-go.

The complexity of the inner loop on a network *G* of size *n* can be estimated as follows. The repetition of the loop changes as *R* is updated. The list of neighbours (i.e., *R*) initially starts with the neighbours of node *v*. Let us assume *k* is the average degree of *G*. In this case, The initial size of *R*, in other words, the repetition of the first loop is *k* (*t*_1_ = *k*). As *R* progressively is extended by adding other nodes, the next loop repetitions *t*_2_, *t*_3_, …, *t*_*m*_ increases as well. To calculate the complexity, we need to sum up all recurrences of the loop: {*t*_1_ = *k*, *t*_2_ = *k*^2^, …, *t*_*m*_ = *k*^*m*^}. Considering the size of the network, the final *R* includes all nodes of *G*, therefore, *t*_*m*_ = *k*^*m*^ = *n*. Then, the complexity of the series that is combining the loops is *O*(*t* × *n*), with *t* representing the iterations over the outer *while*-loop. In addition, according to our experiments [29] *t log*(*n*), hence the average-case complexity of lda-go is in the order of *nlog*(*n*).

The worst-case scenario happens when the inner-loop runs over *V* instead of *R*. In this case, each iteration performs on *n* nodes instead of *k*. The recurrence of the inner-loop is then, {*t*_1_ = *n*, *t*_2_ = *n*, …, *t*_*m*_ = *n*}. However, the iterations of outer-loop remains the same since it is independent from the inner-loop. Hence, the worst case complexity stays as same as the average complexity, *O*(*nlog*(*n*)).

## Experiments and results

In this section, we first describe the ppi network dataset, go [12] terms that are used to enrich the network, and the benchmark dataset. Next, we define the metrics and measures that we use to evaluate the performance of our algorithms, lcda and lcda-go. Finally, we provide a comparative evaluation to show the performance of our algorithm compared to state-of-the-art algorithms.

### PPI network and Gene Ontology (GO)

To evaluate lcda-go and lcda, Krogan [40] dataset is selected. It includes a set of nodes (i.e., proteins) and associated links (i.e., interactions) built on yeast *Saccharomyces Cerevisiae* data. We download the dataset from BioGrid database [41]. To include the functionality we exploit Gene Ontology (go) terms from Panther database [42]. go terms are subdivided into three categories of Molecular Function (mf), Biological Process (bp) and Cellular Component (cc). We extract the go terms of Krogan ppi network. For evaluating the outcome, we use gold standard protein complexes cyc2008 [43] as target sets to evaluate the predicted communities resulted from lcda-go. The information associated with the database and datasets are described in Table 2.

**Table 2:** Datasets of networks used for the experiments.

PPI Network					
Datasets	|*V*|	|*E*|	avg. degree	# CC	|*G*_*cc*_|
Krogan [40]	2674	7079	5.29	62	2527
PPI + MF	1014	2135	4.21	7	995
PPI + BP	1154	2502	4.33	8	1130
PPI + CC	1160	2710	4.67	10	1130
PPI + All	1523	3708	4.86	9	1498
Gene Ontology (GO)					
Database	Proteins	# MF functions	# BP functions	# CC functions	All functions
Panther [42]	2358	8	11	3	22
Benchmark					
Database	Proteins	Complexes	# ∩ Krogan	# ∩ Panther	
CYC2008 [43]	1920	408	970	813	

Krogan ppi network [40] dataset, includes 2674 proteins in total. Our analysis found 62 connected components with a giant connected component including 2527 proteins, while 42 of the components had less than 3 nodes. For the community detection, we removed all those 42 components that will not shape a community. The final ppi network includes 2590 proteins.

We generated four ppi networks from the original Krogan ppi network according to go term categories: ppi + mf, ppi + bp, ppi + cc, ppi + all, such that the last network includes all the functions. We also keep the original Krogan network without annotations for further analysis. All five networks are refined by filtering the connected components with the size of less than 3 proteins.

### Evaluation metrics

Before presenting the evaluation results, we describe various metrics that are mostly used in the literature [2, 23, 31, 32] to assess detected complexes in ppi networks. Exploiting these metrics, we then compare the state-of-the-art algorithms with our proposed algorithm and describe them.

#### Neighbour affinity score

To quantify the similarity of the detected complex *p* = (*V*_*p*_, *E*_*p*_) with the benchmark *b* = (*V*_*b*_, *E*_*b*_), we use the neighbour affinity score (*AS*) as defined in Eq 4. This metric considers both the size of the two complexes and the common proteins in the two sets to measure the similarity between the two. In case the predicted complex is exact equal to the real complex, then *AS* will be equal to 1. For two complexes of *p* and *b* the affinity score is defined as follows:

$$\begin{array}{c} \begin{array}{c} AS\left(p,b\right)=\frac{|V_{p}\cap V_{b}{|}^{2}}{|V_{p}\left|.\right|V_{b}|} \end{array} \end{array}$$ (4)

where *V*_*p*_ is the number of proteins from the predicted complex and *V*_*b*_ is the number of proteins in the benchmark complex. We define a threshold *θ*, *AS*(*p*, *b*) ≥ *θ*, to control the strength of the similarity measured by AS. We consider *θ* = 0.1 to get results from all algorithms.

#### Precision, recall, and F-measure

Among the standard metrics to evaluate the predicted values based on the benchmark are *Precision*, *Recall*, and *F* − *measure*. However, the metrics that we have implemented in this paper for the evaluation are slightly different than the common definition for the *Precision*, *Recall*, and F-measure and are similar to [2, 44]. We use *AS* as defined in Eq 4 to choose a good match between the predicted and benchmark complexes. Assume that *p* is a predicted complex from the set of all predicted complexes *P*, and *b* is a benchmark complex from set *B* that includes all benchmark complexes. In this case, *N*_*cp*_ and *N*_*cb*_ are defined as follows:

$$\begin{array}{cc} N_{cp}= & |\{\forall p|p\in P,\exists b\in B,AS(p,b)\geq \theta \}|,\\ N_{cb}= & |\{\forall b|b\in B,\exists p\in P,AS(p,b)\geq \theta \}|. \end{array}$$ (5)

Based on the *N*_*cp*_ and *N*_*cb*_ values from Eq 5, Precision, Recall are defined as the fraction of the matched complexes from the predicted set *P*, and benchmark set *B* respectively, according to the Eq 6.

$$Precision=\frac{N_{cp}}{\left|P\right|},$$ (6a)

$$Recall=\frac{N_{cb}}{\left|B\right|}.$$ (6b)

The harmonic average of *Precision* and *Recall*, known as F-measure, is then calculated as follows:

$$\begin{array}{c} F-measure=\frac{2\times Precision\times Recall}{Precision+Recall} \end{array}$$ (7)

We use these metrics to evaluate the overall performance of the detected complexes over the complexes within the benchmark.

#### Sensitivity, positive predicted value, and accuracy

Besides the metrics defined above, *Sensitivity* (*Sn*) (also called *Coverage*), *Positive Predicted Value* (*PPV*), and *Accuracy* (*Acc*) are used to evaluate the performance and accuracy of the detected complexes [2, 9, 32]. Consider *T*_*ij*_ equal the number of common proteins between *i*^*th*^ benchmark complexes and *j*^*th*^ predicted complex. *N*_*i*_ is the number of proteins the *i*^*th*^ benchmark complex. Given *n* is the overall number of *b* benchmark complexes and *m* predicted complexes *p*, then *Sn* and *PPV* are defined as follows:

$$Sn=\frac{\sum_{i=1}^{n}{\text{max}}_{j}\left(T_{ij}\right)}{\sum_{i=1}^{n}N_{i}},$$ (8a)

$$PPV=\frac{\sum_{j=1}^{m}{\text{max}}_{i}\left(T_{ij}\right)}{\sum_{j=1}^{m}\sum_{i=1}^{n}T_{ij}}.$$ (8b)

Larger values of *Sn* indicate that the community detection algorithm has well-covered the proteins in the real complexes. On the other hand, *PPV* highlights the probability of true positives of protein complexes in predicted communities. The accuracy of the prediction, as a summary metric, can then be defined as as the geometric average of *Sn* and *PPV* as follows:

$$\begin{array}{c} Acc=\sqrt{Sn\times PPV} \end{array}$$ (9a)

In addition to the above-mentioned metrics, several studies [23, 35, 45] rely on another measure known as *Composite Score* [46] to make a comprehensive evaluation. Therefore, as a final global performance measure, we calculate the *Composite Score* by summing up the three values of *Precision*, *Sn*, and *Acc*. This value is important to avoid the advantage of evaluation metrics to another.

### Comparative evaluation

We provide a set of experiments to compare the communities resulted from our algorithm with the state-of-the-art algorithms. We compared lcda-go and lcda [29] with mcode [33], mcl [34], ClusterOne [35], and mtgo [23]. We choose these algorithms to explore the benefits of topological and functional properties in the performance of protein complex detection methods.

Except our two algorithms, lcda and lcda-go, other algorithms require setting up initial parameters such as *min size* of the community, in their software. Clearly, tuning the parameters could result in better performance, however, there is no principled way to discover the optimal values for these parameters rather than using their defined values. Table 3 describes a general overview of the results of employing different community detection algorithms on ppi networks. In all experiments, we benefit from the gold standard protein complexes of cyc2008 [43] as the benchmark.

**Table 3:** An overview of the resulted communities from each algorithm including our method on *Saccharomyces Cerevisiae* Krogan interaction datasets.

PPI + MF					
Algorithms	MCODE	MCL	ClusterOne	LCDA	LCDA-GO
#communities	37	244	209	65	383
*N*_*cb*_	4	160	142	69	167
*N*_*cp*_	2	112	117	36	154
PPI + BP					
Algorithms	MCODE	MCL	ClusterOne	LCDA	LCDA-GO
#communities	38	256	236	71	416
*N*_*cb*_	3	192	170	76	202
*N*_*cp*_	3	149	146	51	196
PPI + CC					
Algorithms	MCODE	MCL	ClusterOne	LCDA	LCDA-GO
#communities	51	277	237	71	425
*N*_*cb*_	6	196	180	80	210
*N*_*cp*_	5	158	153	54	211
PPI + All					
Algorithms	MCODE	MCL	ClusterOne	LCDA	LCDA-GO
#communities	52	347	142	79	548
*N*_*cb*_	4	213	122	78	223
*N*_*cp*_	4	178	106	52	237

To provide fair comparisons and for a detailed analysis, we have designed two experiments. In the first experiment, we only consider the communities that are detected by the algorithms only considering the topology of the network, namely, mcode [33], mcl [34], ClusterOne [35], lcda [29]. The second experiment is for evaluating the communities resulting from algorithms that are incorporating both topology and functionality. For this evaluation, we compared lcda-go with mtgo [23]. The next two subsections present the comparisons of these experiments.

#### Topological algorithms analysis

**Fig 2:**
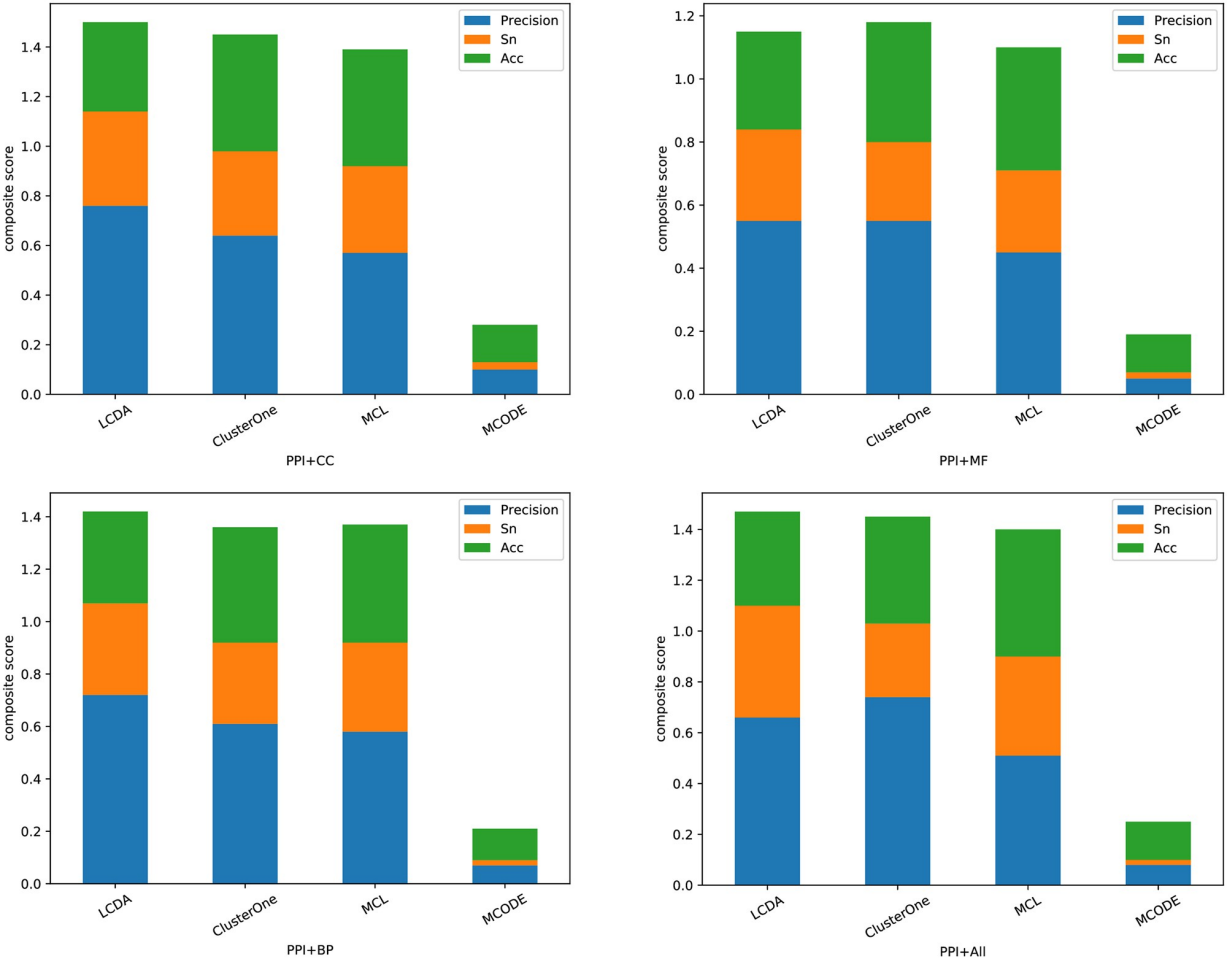
Composite score including *Precision*, *Sn*, and *Acc*.

We compare our lcda [29] algorithm that solely considers the topological interaction of the ppi network with other algorithms from the literature that perform in a similar manner. We select mcode [33], mcl [34], and ClusterOne [35] for this comparison. We have used Cytoscape software [47] and exported the communities resulted from these methods. The input networks are extracted from Krogan dataset and divided based on go functionalities. The assessments are described for all four algorithms in Table 4 based on the metrics explained earlier in this section. As presented in the table, the performance of mcode is considerably low compared to the other algorithms, even though we have set *θ* = 0.1 to relax the condition for *AS*. mcl has overall the highest *Recall*, *Fmeasure*, and *Acc*, while our lcda algorithm outperforms other algorithms with the highest *Precision*, *Sn*, and particularly *Composite Score*. The performance of ClusterOne algorithm is also high and relatively close to both MCL and and our algorithm lcda. The *Composite Score* is shown in Fig 2. The total height of each bar is the value of the *Composite Score* and the larger scores are better. The figure describes how the three algorithms are competing for a higher performance rank and lcda is outperform them.

**Table 4:** Performance comparison of the communities of the algorithms that are based on only topology on *Saccharomyces Cerevisiae* Krogan interaction datasets. *θ* is 0.1.

PPI + MF							
Algorithms	*Precision*	*Recall*	*F*-*measure*	*Sn*	*PPV*	*Acc*	*Composite Score*
MCODE	0.05	0.01	0.02	0.02	**0.65**	0.11	0.19
MCL	0.45	**0.39**	**0.42**	0.26	0.60	**0.39**	1.11
ClusterOne	**0.55**	0.35	**0.42**	0.25	0.58	0.38	**1.19**
LCDA	**0.55**	0.16	0.26	**0.29**	0.33	0.31	1.16
PPI + BP							
Algorithms	*Precision*	*Recall*	*F*-*measure*	*Sn*	*PPV*	*Acc*	*Composite Score*
MCODE	0.07	0.00	0.01	0.02	**0.68**	0.12	0.22
MCL	0.58	**0.47**	**0.52**	0.34	0.62	**0.45**	1.38
ClusterOne	0.61	0.41	0.49	0.31	0.63	0.44	1.37
LCDA	**0.72**	0.17	0.30	**0.35**	0.35	0.35	**1.41**
PPI + CC							
Algorithms	*Precision*	*Recall*	*F*-*measure*	*Sn*	*PPV*	*Acc*	*Composite Score*
MCODE	0.10	0.01	0.02	0.03	**0.78**	0.15	0.28
MCL	0.57	**0.48**	**0.52**	0.34	0.65	**0.47**	1.39
ClusterOne	0.64	0.44	**0.52**	0.34	0.63	0.46	1.45
LCDA	**0.76**	0.20	0.31	**0.38**	0.34	0.36	**1.50**
PPI + All							
Algorithms	*Precision*	*Recall*	*F*-*measure*	*Sn*	*PPV*	*Acc*	*Composite Score*
MCODE	0.08	0.01	0.02	0.03	**0.75**	0.15	0.26
MCL	0.51	**0.52**	**0.51**	0.39	0.63	**0.50**	1.40
ClusterOne	**0.74**	0.30	0.45	0.30	0.60	0.42	1.46
LCDA	0.66	0.20	0.30	**0.44**	0.31	0.37	**1.47**

#### Topological and functional algorithms analysis

We implement and test our proposed algorithm for protein complex detection, lcda-go on all the networks extracted from Krogan dataset. The results are described in Table 5.

**Table 5:** Performance of lcda-go on *Saccharomyces Cerevisiae* from Krogan interaction datasets.

Network	*Precision*	*Recall*	*F*-*measure*	*Sn*	*PPV*	*Acc*	*Composite Score*
PPI + MF	0.40	0.41	0.41	0.19	0.62	0.35	0.94
PPI + BP	0.72	0.17	0.30	0.35	0.35	0.35	1.41
PPI + CC	0.50	0.51	0.51	0.27	0.64	0.41	1.17
PPI + All	0.43	0.55	0.48	0.28	0.65	0.43	1.15

**Fig 3:**
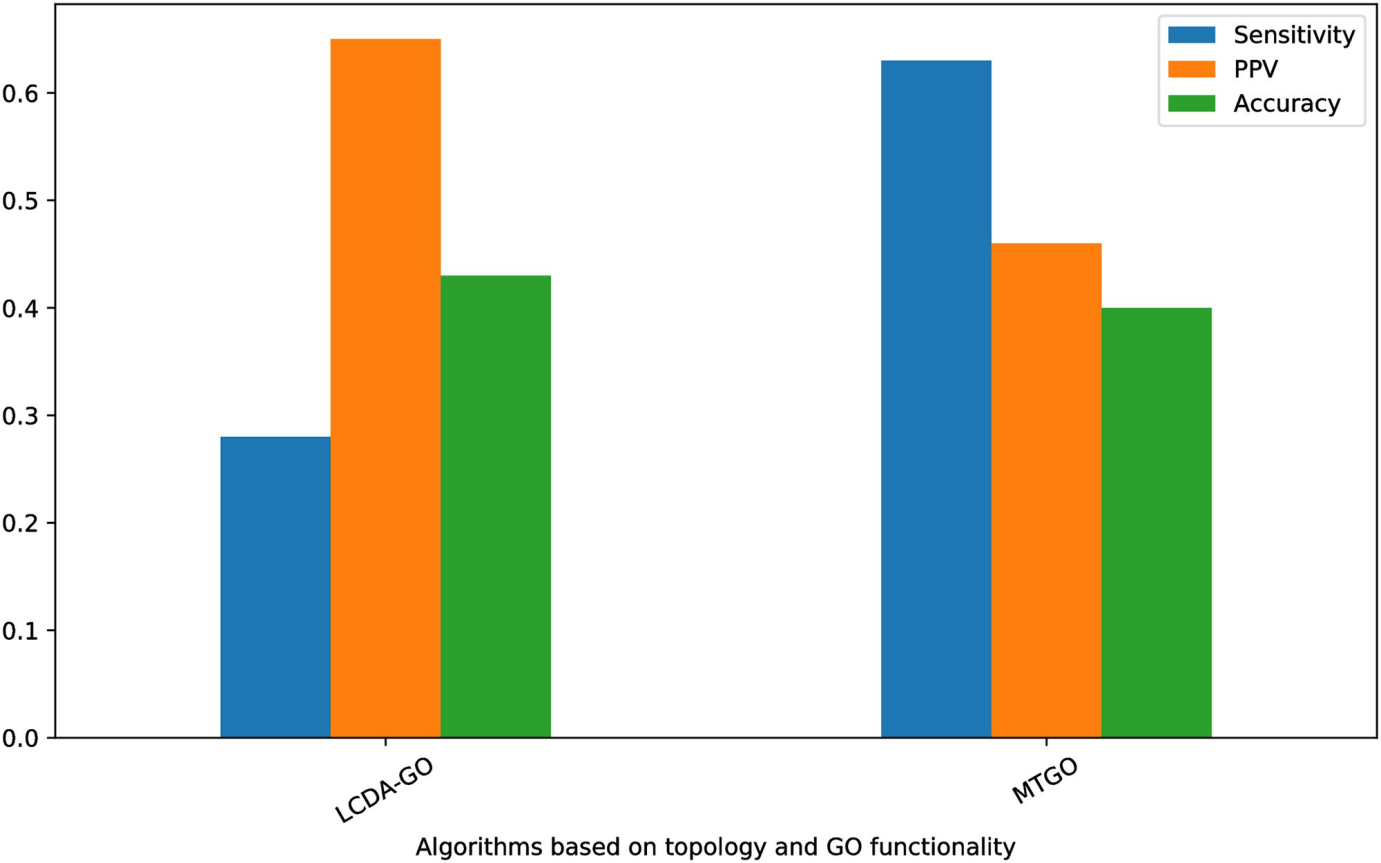
Comparing the results of lcda-go with mtgo on Krogan dataset.

We choose mtgo to compare the results of lcda-go with since it also considers functionality as a parameter involved in the community detection and not as an in dependant process that could apply after community detection algorithm. We have exploited the mtgo software to run over the Krogan networks from Table 2, however, considering the large time complexity of this algorithm the final results could not converge by the time of writing this paper. Therefore, we decided to rely on the experiments attached to their studies for this comparison. We choose only *Sn*, *PPV*, and *Acc* to compare the results due to the fact that they are independent from the threshold required for *AS* score. The results are presented in Fig 3. As shown in this figure, even though mtgo has better *Sn* compared to lcda-go, *PPV* and *Acc* of lcda-go is larger. Overall, the two algorithms are competitive based on these assessments.

#### Computational complexity analysis

Besides, the relatively close results from lcda-go and mtgo is the complexity of the two algorithms. Due to the locality of lcda-go, our algorithm enjoys from the loglinear time complexity while mtgo is a polynomial time algorithm. Our algorithm is more than 1400 times faster than mtgo when performing on Krogan dataset with 2674 nodes. The time complexity of lcda-go and mtgo is compared in Table 6.

**Table 6:** Complexity and run time of algorithms incorporating go on Krogan network.

Algorithm	Time (sec)	Complexity
LCDA-GO	47.05	*O*(*nlog*(*n*))
MTGO	54000	*O*(*kn*^3^)

## Discussion and conclusion

Identifying protein complexes is an important step for biological knowledge discovery since several biological processes are accomplished in the formation of protein complexes. In this paper, we propose a local community detection algorithm, lcda-go, for protein complexes by exploiting Gene Ontology (go). lcda-go exploits networks’ topological properties such as degree and shortest path in conjunction with protein’s functional properties derived from go databases. Our algorithm employs both topological and functional properties in local measures to perform on ppi networks in a local procedure.

We evaluate lcda-go and another variation of the algorithm called lcda, the latter relying only on the topology of the network. Experimental results demonstrate their performance on real-world ppi networks from the Krogan dataset and their capabilities in finding protein complexes.

In addition, the promising performance of lcda and lcda-go show the capability of our algorithms in successfully detecting protein complexes in ppi network with significantly lower time complexity than the state-of-the-art. lcda-go surpasses the state-of-the-art algorithms by performing on a log-linear time complexity, while recent algorithms such as mtgo run on polynomial time complexity.

One of the limitations of lcda-go is that it can only discover networks including one connected component. The algorithm relies on breadth-first search to discover the network, it thus could not converge if the network consists of more than one connected components. One solution to avoid this issue is to identify the connected components of the network before executing lcda-go and provide one node from each component as the input for the algorithm.

To extend our algorithm, we plan to evaluate lcda-go from functionality aspects. A go term analysis could provide an evaluation on the significance of the functions within each community. Moreover, considering the various attributes utilized in ppi networks, we plan to analyze ppi networks from *attributed network* [48] prospect. We believe that the algorithm could expand for applications in the context of attributed networks.
